# Indoor air quality and early detection of mould growth in residential buildings: a case study

**DOI:** 10.14324/111.444/ucloe.000049

**Published:** 2022-11-15

**Authors:** Arianna Brambilla, Christhina Candido, Ozgur Gocer

**Affiliations:** 1 School of Architecture, Design and Planning, The University of Sydney, Sydney, Australia; 2 Faculty of Architecture, Building and Planning, The University of Melbourne, Melbourne, Australia

**Keywords:** mould growth, hygrothermal, indoor environment, health, sustainability, indoor air quality

## Abstract

Mould growth affects one in three homes, and it is the biggest cause for complaints and litigations filed to the relevant authorities in Australia, while also significantly affecting the physical and psychological health of the building’s occupants. Indoor mould is caused by excessive dampness, resulting from poor architectural specification, construction and maintenance practices, as well as inappropriate behaviour of the occupants. The consequences range from early biodeterioration of building materials, requiring anticipated renovation works, to deterioration of the indoor environment, posing a serious threat to the building’s occupants. This study investigates indoor air quality (IAQ) and mould growth, providing a snapshot of the current IAQ of Australian residential buildings regarding air pollutants. It uses a case study representative of the typical Australian suburban home to investigate the effects of unnoticed mould growth. The results of the monitoring campaign indicate that buildings with a high concentration of fungal spores are also more likely to present poor IAQ levels, high concentrations of particulate matters (PM_10_ and PM_2.5_) and carbon dioxide (CO_2_). This research suggests the need for the development of early detection strategies that could minimise the health hazard to people, thereby preventing the need for any major renovations.

## Introduction

Considering that humans spend a substantial amount of time indoors, indoor air quality (IAQ) has the potential to significantly impact people’s health and wellbeing [1,2]. The IAQ contributing factors can be divided into physical parameters, such as temperature and humidity; chemical parameters, such as concentration of pollutants and substances in the air; and biological parameters, which include the presence of organic compounds and mould [3–5]. The prevalence of indoor mould growth is further amplified by inappropriate design strategies [6], poor construction and maintenance practices [7,8] and occupants’ living conditions [4,8,9] that may lead to indoor accumulation of moisture or condensation within the envelope [10]. Despite its great influence, mould growth is seldom considered nor well specified in building codes [4], and the lack of a standard makes it difficult to establish its exact prevalence and diffusions.

Excessive dampness and indoor mould are estimated to affect between 10% and 50% of the global building stock, a percentage that is likely to be even higher in less privileged communities making exposure to mould a significant risk factor for individuals and communities [6]. Indeed, the digesting process of fungi growing within the building envelope reduces the building’s materials and the components’ service life [11], and the early biodeterioration requires anticipated and extensive renovation works, with the consequent economic loss [8,9,12,13]. Furthermore, long exposure to indoor mould can have adverse health impacts with different degrees of severity. Generally, asthma appears to be the most diffused symptom of exposure [14], with an estimate indicating mould as being responsible for 21% of the confirmed asthma cases in the United States [15], followed by other forms of allergies, such as pneumonitis, alveolitis, allergic rhinitis and sinusitis [12,16,17]. In general, indoor mould affects the psychological wellbeing of the occupants of affected buildings, who can experience a sense of fatigue and reduction of the capacity to concentrate [18], with cases escalating to cognitive impairment and reduced productivity at work [18,19]. These issues are further amplified by the difficulty of detecting mould before it is fully germinated [20], highlighting the importance of early detection of the presence of indoor mould.

In Australia, one in three homes suffers from mould growth [20] with evidence indicating that design and construction practices, systematically, do not properly consider moisture and moisture-related issues [4,21]. For example, the National Construction Code (NCC) introduced hygrothermal provisions for the envelope design of buildings for the first time in 2019 [22] and with a specific focus on residential new construction only. These new provisions, coupled with the increasingly stringent thermal and airtight requirements, developed as energy efficiency measures to respond to the international call for action against climate change, contributed significantly to the rapid rise of indoor mould cases in newly built homes [4,13]. On the other hand, older buildings are prone to mould growth due to poor thermal insulation and the presence of thermal bridges [8], making mould an issue that transcends the age of a building’s construction. These inherent issues of the Australian construction industry are further magnified by the repercussions of climate change and the impacts of the global pandemic. On the one hand, increased levels of humidity, warmer summers, more frequent extreme weather events, such as flooding and prolonged rainy periods create favourable conditions for mould to grow. On the other hand, building occupants spending more time at home are both increasing the indoor moisture generation [23] and their exposure time, posing a serious health threat to themselves. This is even more pronounced within vulnerable communities, rentals and social housing due to a combination of poor building and environmental performance and financial limitations and control over remediation [4].

This study aims to explore the correlations between air pollutants, IAQ and biological growth in the Australian context. The ultimate goal is to develop a better understanding of possible indicators of mould presence that can be diffusely used for early detection, which, in turn, may prevent serious health implications and significant economic loss.

## Methods

This scoping study undertakes an empirical investigation of a case study conducted on a residential building, which can be considered as the typical Australian dwelling. The investigation included a site inspection, air testing and surface sampling for mould detection, as well as monitoring a 2-month long IAQ campaign (during winter). This analysis has been prompted by adverse health symptoms observed in one building occupant, which were not followed by clinical reasons.

### Case study

**Figure 1:**
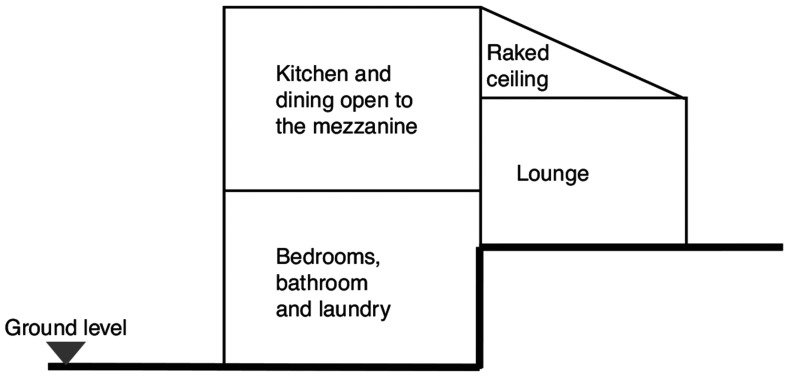
Diagram of the vertical distribution.

The case study is a two-storey residential building located in Gowrie, a suburb of Canberra, ACT, Australia. The dwelling is a typical reversed brick veneer with a timber frame, and it can be considered a good representation of the average Australian home. More than two thirds of Australians live in similar single family or semi-detached town houses [24], making this case study highly representative of a bigger cohort of buildings. This building was built before 1988 and, at the time of the investigation, did not undertake any major renovation. The architecture of the building comprises a split level with high raked ceilings and a mezzanine level, four bedrooms and two bathrooms. The bedrooms, bathrooms, laundry and kitchen face south with no direct solar entrance (southern hemisphere), while the lounge and entry face the north, as represented in Fig. 1. As is commonly found in average Australian homes, no mechanical ventilation, heating or conditioning system was present. However, a closed fireplace is located between the kitchen and the lounge.

Canberra, where the building is located, is characterised by an oceanic climate [25], where the warmest month has a mean maximum temperature of 30.6°C but can register temperatures around 0°C with frequent frost during July (Table 1), when the mean number of days with temperatures below zero is 16.

**Table 1.** Monthly mean temperature in Canberra [25]

	Jan	Feb	Mar	Apr	May	Jun	Jul	Aug	Sept	Oct	Nov	Dec
Mean max temp [°C]	30.6	28.1	25.0	21.0	16.6	13.4	12.7	14.1	18.0	21.5	24.9	27.5
Mean min temp [°C]	14.2	13.5	11.1	6.8	2.4	1.2	0.1	0.9	3.1	6.2	9.8	12.1

This area is also characterised by frequent precipitation. The annual average rainfall is approximately 700 mm, with the highest intensity registered in October (66 mm) and the lowest in June (38.1 mm) [25]. On average, there are around 100 days of precipitation per year, with July being the wettest month (11 days of precipitation) and April the driest (6 days) [25].

### Monitoring campaign and sampling procedures

The experimental campaign involved inspections, mould detection and IAQ monitoring [26].

The initial inspection took place in June 2020, and it was aimed at detecting potential risk sources and understanding occupancy habits to inform the monitoring campaign, which started in early July 2020. Table 2 shows the parameters monitored during the experimental campaign. It is worth mentioning that occupants were asked to conduct their normal activities during the monitoring phase, in order to gather information representative of their everyday environmental conditions.

**Table 2.** IAQ monitoring equipment

Parameter	Sensor type	Accuracy	Resolution	Range
PM_10_; PM_2.5_	Light scattering (350 nm)	±10% (<30 μg/m^3^ ±3 μg/m^3^)	1 μg/m^3^	1/100030 μg/m^3^
CO_2_	Non-dispersive infrared	±3% ±50 ppm	1 ppm	400/2000 ppm
TVOC	MOS	±15%	1 ppb	N/A
Temperature	Digital	±1°C	1°C	−200/100°C
Relative humidity	Digital	±5%	1%	0/99%

During July, several surfaces and air samples were taken around the house with the scope of identifying potential fungal strains [26]. For this research, off-the-shelf instruments for mould sampling were used, following the standard protocols described in the device’s instruction manuals, which were compared against the literature [26] to ensure consistency.

The surface samplings were collected using Zefon adhesive Bio-Tape slides (Zefon International, Ocala, Florida, US), which consist of a plastic slide with a pre-adhesive area of 4 cm^2^ that must be gently pressed on the designated surface before returning the slide to its individual protective case. This bio-tape allows determining the presence of microbial, bio-aerosol and inorganic dust, identifying the mould strains and quantifying the degree of contamination [26]. The sampling locations were selected after the inspection, as per Portnoy et al. [26] and the manufacturer’s instructions.

Air sampling used Air-O-Cell cassettes (Zefon International, Ocala, Florida, US), which are based on inertial impaction (the cassette is provided with a tapered inlet that directs particulate-laden air towards a slide with the collection media). This cassette falls under the sampler type ‘impaction onto coated surface’ [26], where the collecting media is a cellulose acetate-coated glass. The sampling location was determined by the site inspection, as, following the standard protocol, it was performed near the centre of each area of the buildings affected by moisture intrusion, water damage, apparent mould growth, musty odours and conditions conducive to mould growth. Following the manufacturer’s instructions, the cassettes were operated with a flow rate of 15 l/m and activated for 10 minutes.

The sampling devices were characterised by a detection limit of 38 FS/m^3^ and of 4 FS/m^3^, respectively, for air and surface sampling.

All samples were then analysed by a certified laboratory, following the standard protocols and the manufacturer’s instructions, staining the sample with lactophenol cotton blue and a professional magnifier for microscopic examinations.

## Results and discussion

### Site inspections

During the initial inspection of the building, several risk factors for mould growth and poor IAQ were noticed. The lower level was characterised by poor lighting, with very low daylight levels. This space was designed to be lit by the windows in the bedrooms, which were, however, always kept closed and equipped with sunblock curtains, as they were not occupied. Furthermore, this contributed to reducing minimum natural cross ventilation throughout the lower level, where wet and humid rooms, such as bathrooms and the laundry, are also located and, consequently, preventing moisture laden air being correctly exhausted by those rooms. This situation was aggravated by a leaky wall cavity from the shower, indicated by the damp odour originating from the bathroom, visible water staining on the adjacent walls and damp-stained furniture laid against the wet wall. All these details indicated that indoor mould was likely to be found in this area of the dwelling. Based on the inspection, the surface samples were taken from the visible stains (bedroom wall and hallway cupboard), as well as from the kitchen bench as a control sample for an area not visibly affected.

The mould sampling results reveal that indoor mould was highly diffused in the building, as indicated in Table 3.

**Table 3.** Results of the mould sampling (expressed in FS/m^3^)

Sample		Total fungal structures	Mould genera	
Air sample	Kitchen	3994	*Aspergillus/Penicillium*	2765
			*Basidiospores*	499
			*Cladosporium*	77
			*Smut/Myxomyces/Periconia*	269
	Hallway	5760	*Alternaria*	38
			*Aspergillus/Penicillium*	2918
			*Basidiospores*	998
			*Cladosporium*	154
			*Curvularia*	38
			*Spegazzinia*	154
	Bedroom (occupied)	9638	*Aspergillus/Penicillium*	7680
			*Basidiospores*	806
			*Bipolaris/Drechslera*	38
			*Cladosporium*	154
			*Oidium/Peronospora*	38
			*Smut/Myxomyces/Periconia*	192
Surface swab	Kitchen bench	83	*Ascospores*	8
			*Aspergillus/Penicillium*	75
	Hallway cupboard	17	*Aspergillus/Penicillium*	17
	Bedroom wall (occupied)	46	*Aspergillus/Penicillium*	38
			Basidiospores	8

Results identify Aspergillus spp. as the predominant mould genera, which confirms the literature [4]. This fungal strain is among the most common, yet one of the most critical for human health [12], being responsible for various insidious infections and correlated to sick building syndrome [27]. *Cladosporium* is more commonly found on exterior facades [4] and its detection indoors suggests a potential exchange of spores from the outside. Nonetheless, at least 24 different fungal strains were detected, of which 50% are usually regarded as a source of respiratory infections, 21% are able to produce mycotoxins, which constitutes a second health hazard, and 25% are usually considered a proxy for condensation issues. As expected, the bedrooms and the hallway presented a higher concentration of spores, due to the high humidity accumulated indoors by poor ventilation practices and water leakages in the envelopes. Unexpectedly, the kitchen also showed elevated spore concentrations, albeit lower than the other rooms. The concentration found in the kitchen may be originated from other organic sources, such as food remnants developing mould, however, during the sampling, no such condition was observed and the occupants, when asked about this possibility, confirmed the low probability of this option. These results indicate that the effects and implications of indoor mould may affect a much larger area than the affected surface, mainly due to the volatile nature of spores and mycotoxins combined with a higher cleaning frequency of surfaces with antibacterial products. In this case, mould was expected in the unoccupied rooms, but effectively found across the whole dwelling, determining a high risk for the occupants.

### Indoor air quality *monitoring and sampling*

The indoor environmental parameters were monitored between 28 June and 9 September 2020. Acceptability thresholds for indoor pollutant concentrations have been considered as shown in Table 4.

**Table 4.** Measured PM_10_, PM_2.5_, CO_2_ and TVOC values and Indoor Air Quality thresholds [28]

Parameter monitored	Acceptable threshold	Note
PM_10_	50 μg/m^3^	Averaged on 1 h
PM_2.5_	25 μg/m^3^	Exceptional event rule: an exceptional event is a fire or dust occurrence that adversely affects air quality at a particular location; causes an exceedance of one 1-day average standards in excess of normal historical fluctuations and background levels, and is directly related to bushfire, jurisdiction-authorised hazard – reduction burning or continental-scale windblown dust
CO_2_	850 ppm	Averaged on 8 hours. NCC IAQ Verification Method This level is based on an increment of 450 ppm above the background CO_2_ concentration, representing an adequately ventilated building
TVOC	500 μg/m^3^	Averaged on 1 h

**Figure 2:**
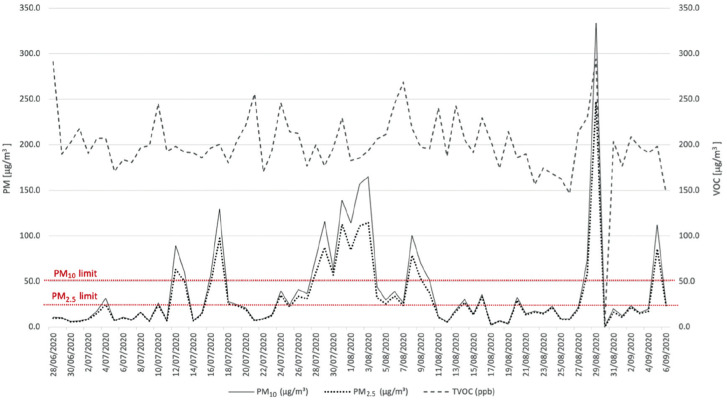
Monitored values of PM_10_, PM_2.5_ and TVOC.

Figure 2 shows the results obtained from monitoring of indoor particulate matter (PM_10_, PM_2.5_) and total volatile organic compound (TVOC) values. Based on the defined acceptability limits (Table 4), the IAQ analysis shows that concentrations of pollutants have been consistently higher than the expected thresholds, especially during the periods of 13/07, 17/07/, 29/07, 01/08–04/08, 09/08 and 29/08. A similar general pattern is exhibited for both PM_2.5_ and PM_10_ values. The differences between the defined threshold and measured PM values were approximately 225 μg/m^3^ and 280 μg/m^3^ for PM_2.5_ and PM_10_ values, respectively. The peak values observed in the measurements overlap with the rainy days recorded during those periods [25, weather data from the closest weather station Tuggeranong AWS]. Days with high rainfall over 10 mm were 13, 26–27 July and 8–9 August. However, no rainfall was registered during 29/08, hence the peak indoor measurements are due only to indoor pollutants.

The TVOC level was always lower than the defined threshold (500 μg/m^3^) [29] for the whole duration of the monitoring campaign, ranging between 150 and 250. The peak was registered on 29/08, showing a similar trend observed for PM_2.5_ and PM_10_.

**Figure 3:**
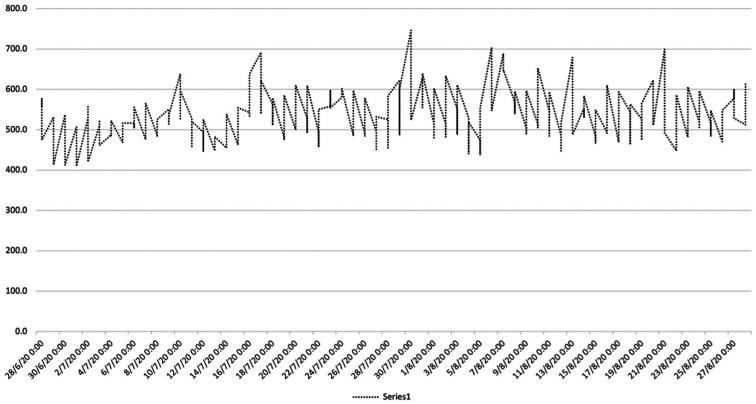
Monitored values of CO_2_ (from 29/08 to 31/08 sensors not working).

Figure 3 displays the monitored values of carbon dioxide (CO_2_) (28 June and 9 September 2020). Except for the first week of monitoring (between 28/06 and 06/07), the CO_2_ values most likely ranged between 500 and 600 ppm which is below the defined threshold for residential buildings [29]. However, some peak measurements that reached the level of 700 ppm were also observed. Considering that the four-bedroom house was occupied by only two residents, excluding the risk of overcrowding, and the observation made during the inspection, this might suggest that the space was significantly under-ventilated and that these parameters may be considered good indicators for early detection of high moisture-related risk probability.

**Figure 4:**
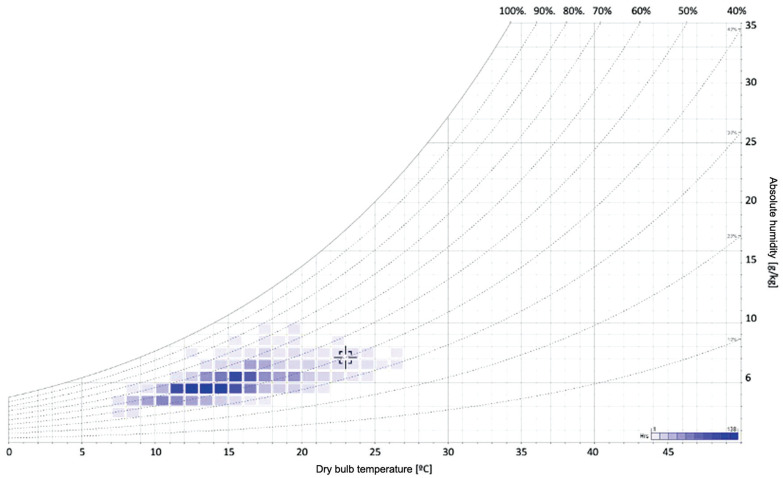
Psychrometric chart with indication of the frequency of values monitored.

Figure 4 displays the monitored temperature and relative humidity on a psychrometric chart. The intensity of the colour in each band represents the frequency of that value, meaning that the darker the block is, the more often that combination of relative humidity and temperature was measured.

Figure 4 reveals that the indoor temperature was consistently below 18°C (80% of the time), with peaks lower than 14°C (42% of the time). Despite that for 81% of the time the indoor relative humidity was in the optimal range of 40–70%, the low temperatures, coupled with cyclical humidity variations and water availability on the surface, favoured mould germination and growth.

## Conclusions

The investigation shows that the extensive mould infestations may easily go undetected for far too long, which in turn requires significant renovations to completely remove this significant health hazard from damaged premises. This case study suggests that high concentrations of fungal spores are correlated to poor IAQ, high concentrations of particulate matter (PM_10_ and PM*_2.5_*), as well as a high levels of CO_2_. It also indicates that underventilation, poor access to daylight and consistently low indoor temperatures, when combined with pre-existing water leakages, may lead to an extensive biological spread that, in turn, increases the biological contamination of the indoor spaces.

Dwellers are seldom involved in the conversation about how to properly manage indoor environments and how to assess and evaluate the risks associated with occupancy practices (e.g., ventilation of unoccupied spaces or laundry drying in small and unventilated spaces), despite being strategic and easy-to-change aspects that determine mould growth. This case study also illustrates that when a poor building envelope design overlaps with high relative humidity and temperature, mould growth in interior spaces becomes difficult to suppress for the climate in question. Furthermore, this case study underlines the necessity of developing early detection strategies that could minimise the risk for occupants, as well as reduce the cost of repairs. Finally, the findings, even though limited to a case study, further add to the call for a change in Australian regulations and construction practice to address mould at the design stage by establishing a prevention-based approach, rather than remediation.

## Data Availability

The datasets generated during and/or analysed during the current study are available from the corresponding author on reasonable request.
